# A Novel Pathogenic *TUBA1A* Variant in a Croatian Infant Is Linked to a Severe Tubulinopathy with Walker–Warburg-like Features

**DOI:** 10.3390/genes15081031

**Published:** 2024-08-05

**Authors:** Akzam Saidin, Anet Papazovska Cherepnalkovski, Zeeshan Shaukat, Todor Arsov, Rashid Hussain, Ben J. Roberts, Marija Bucat, Klara Cogelja, Michael G. Ricos, Leanne M. Dibbens

**Affiliations:** 1 Epilepsy Research Group, Clinical and Health Sciences, Australian Centre for Precision Health, University of South Australia, Adelaide, SA 5000, Australia; akzam.saidin@mymail.unisa.edu.au (A.S.); zeeshan.shaukat@unisa.edu.au (Z.S.); rashid.hussain@unisa.edu.au (R.H.); michael.ricos@unisa.edu.au (M.G.R.); 2 Novocraft Technologies, Petaling Jaya 46300, Malaysia; 3 Department of Neonatology, Clinic for Gynecology and Obstetrics, Clinical Hospital Center Split, 21000 Split, Croatia; anet.cherepnalkovski@gmail.com (A.P.C.); marija.bucat@gmail.com (M.B.); klara_cogelja@yahoo.com (K.C.); 4 Department of Health Studies, University of Split, 21000 Split, Croatia; 5 Faculty of Medical Sciences, University Goce Delcev in Shtip, 2000 Shtip, North Macedonia; todor.arsov@ugd.edu.mk; 6 Clinical and Health Sciences, Health and Biomedical Innovation, University of South Australia, Adelaide, SA 5000, Australia; ben.roberts@unisa.edu.au

**Keywords:** tubulinopathy, lissencephaly, hypotonia, *TUBA1A*, generalized myoclonus, hypoplastic genitalia, macrocephaly

## Abstract

Tubulinopathies are associated with malformations of cortical development but not Walker–Warburg Syndrome. Intensive monitoring of a Croatian infant presenting as Walker–Warburg Syndrome in utero began at 21 weeks due to increased growth of cerebral ventricles and foetal biparietal diameter. Monitoring continued until Caesarean delivery at 34 weeks where the infant was eutrophic. Clinical assessment of a progressive neurological disorder of unknown aetiology found a macrocephalic head and markedly hypoplastic genitalia with a micropenis. Neurological examination showed generalized hypotonia with very rare spontaneous movements, hypotonia-induced respiratory insufficiency and ventilator dependence, and generalized myoclonus intensifying during manipulation. With clinical features of hypotonia, lissencephaly, and brain malformations, Walker–Warburg Syndrome was suspected; however, eye anomalies were absent. Genetic trio analysis via whole-exome sequencing only identified a novel de novo mutation in the *TUBA1A* gene (NM_006009.4:c.848A>G; NP_006000.2:p.His283Arg) in the infant, who died at 2 months of age, as the likely cause. We report a previously unpublished, very rare heterozygous *TUBA1A* mutation with clinical features of macrocephaly and hypoplastic genitalia which have not previously been associated with the gene. The absence of eye phenotypes or mutations in Walker–Warburg-associated genes confirm this as not a new presentation of Walker–Warburg Syndrome but a novel *TUBA1A* tubulinopathy for neonatologists to be aware of.

## 1. Introduction

Tubulinopathies are a heterogeneous group of disorders caused by mutations in members of the tubulin superfamily of genes [1]. Among the five tubulin families, members from the α-α and β-β families encode tubulin proteins which form heterodimers and are two of the main components of microtubules [1]. Tubulins are involved in a range of cellular processes including intracellular transport, cell division, and neuronal migration [1]. While tubulinopathies have broad clinical presentations, many include cortical and subcortical brain malformations such as lissencephaly, polymicrogyria, and cortical dysplasia [1,2]. Other clinical features include microcephaly, global developmental delay, intellectual disability, and seizures [1]. *TUBA1A* mutations are the most common cause of tubulinopathies with neurological phenotypes, and to date, all identified pathogenic *TUBA1A* mutations appear to be autosomal dominant heterozygous loss of function mutations [1].

Walker–Warburg Syndrome (WWS) is a rare autosomal recessive disease where patients present with several comorbid phenotypes including brain malformations (both lissencephaly and cerebellar malformations), eye malformations, and congenital muscular dystrophy [3,4]. To date, the majority of genes associated with WWS, like O-Mannosyltransferase Gene (*POMT1*) [5] and Fukutin-related protein (*FKRP*) [6], have been implicated in causing defects in the glycosylation of proteins mainly of α-dystroglycan encoded by the *DAG1* gene, which is also a cause of WWS [7]. As such, WWS is now considered by many to be, first and foremost, a congenital muscular dystrophy caused by α-dystroglycan glycosylation defects with associated brain and eye abnormalities [7].

We investigated an infant of Croatian origin that was born with severe developmental abnormalities of hypotonia and cobblestone lissencephaly type brain malformations indicative of Walker–Warburg Syndrome (WWS) but lacked the associated eye phenotypes. Clinical investigations revealed hydrocephalus and ventriculomegaly indicative of WWS, but the infant also had macrocephalic features, which are generally atypical of WWS. Genetic investigation of the affected infant and their parents by whole-exome trio sequencing revealed a novel de novo heterozygous *TUBA1A* mutation c.848A>G, p.His283Arg that, based on its predicted effects via in silico analysis and 3D protein modelling, was the most likely cause of the disease phenotypes. Based on the presence of the clinical features of hypertonia and brain malformations, Walker–Warburg Syndrome was initially suspected, but the absence of eye phenotypes and the identification of a mutation in the *TUBA1A* gene, which has not been shown to cause glycosylation defects or WWS, did not support a WWS diagnosis. This ultra rare and pathogenic mutation in the *TUBA1A* gene represents a new *TUBA1A*-associated tubulinopathy caused by a previously unpublished *TUBA1A* mutation and a phenotype that should not be conflated with canonical WWS.

## 2. Materials and Methods

### 2.1. Clinical Investigation

Clinical investigation of the infant was performed at the Clinical Centre Split, Croatia. Ultrasound of the brain was performed using a Siemens Healthcare d.o.o., Zagreb, Croatia, Healthineers Acuson NX3 Elite, ultrasound device with a convex transducer frequency of 7.3 MHz. Brain MRI was performed using a Siemens AG, Erlangen, Germany, Magnetom Symphony 1.5 Tesla device.

### 2.2. Genetic Investigation

Chromosomal analysis of a peripheral blood sample from the infant was conducted using Giemsa trypsin (GTG banding). Trio whole-exome sequencing (WES) was performed on genomic DNA extracted from peripheral blood from the patient and their biological parents. DNA libraries were prepared using SureSelect Human All Exon V7 (Agilent, Santa Clara, CA, USA) and sequenced on the Illumina NovaSeq platform (Illumina, San Diego, CA, USA). NovoAlign [8] was used for reads alignment to human reference genome (GRCh38), and the Genome Analysis Toolkit (GATK) HaplotypeCaller was used for joint variant calling [9]. Variant annotation was performed using SnpEff [10] and SnpSift [11] against dbNSFPv4 [12]. Slivar was used for variant prioritization with various inheritance models and to identify structural deletions in parent–child duos using non-transmission of alleles [13]. There was only a single protein-altering de novo sequence mutation identified, and this was classified according to ACMG guidelines using InterVar [14]. Bidirectional Sanger sequencing of genomic DNA confirmed the presence of the mutation in the patient and its absence in the parents. 

A lollipop plot of non-synonymous mutations from clinvar was generated for the TUBA1A protein (UniProt: Q71U36) using the tool lollipops [15]. A total of 167 non-synonymous mutations were extracted from clinvar, comprising 54 pathogenic, 112 likely pathogenic, 1 likely benign, and 0 benign variants. In an effort to uncover genotype–phenotype relationships, we extracted only the pathogenic and likely pathogenic mutations that had accompanying phenotypic information (150 from clinvar of which a subset of 77 mutations is also reported in Tantry et al. (2023) [16]). We then focused on variants located in the tubulin C-terminal domain to compare the associated TUBA1A phenotypes.

### 2.3. Analysis of Mutation Location in the 3D Structure of Tubulin α-1A Chain Protein in a Complex with Tubulin β-III Protein

We performed protein visualization with the software PyMOL (v2.5.5) [17] on the published structure of recombinant neuronal human tubulin (Tubulin α-1A chain protein in a complex with Tubulin β-III protein), 5JCO [18], identifying the mutated residue of interest. The location of the Histidine (residue 283), which is mutated to Arginine, and its hydrogen bonds were marked/highlighted on the α-Tubulin monomers in the 3D tubulin structure.

## 3. Results

### 3.1. Clinical Investigations

The male patient was the second child from the second pregnancy of young non-consanguineous parents; the mother was 32 years of age and the father 34 at the time of birth. A healthy male sibling was 1.5 years old. The diagnosis of a neurological disorder was made prenatally at 21 weeks’ gestation when enhanced ventriculomegaly and rapidly increasing foetal biparietal diameter was detected during fetal ultrasound. The pregnancy was subsequently closely monitored. Serology for Toxoplasmosis, Rubella, CMV, HSV, and Syphilis were performed and excluded acute infection. Table 1 shows a summary of clinical features. The infant was delivered by elective caesarean section at 34 weeks’ gestation with a birth weight of 2.46 kg (61st percentile), birth length of 48 cm (73rd percentile), and a head circumference of 38.5 cm (>99th percentile) [19]. APGAR scores were 5 after 1 min (1 for each function) and improved to 6 (2 for pulse, 1 for the other functions) after 5 min. The infant was resuscitated using the T-piece resuscitator. After applying 5 initial sustained inflation breaths lasting 1 s each, a positive pressure ventilation was continued for 1 min that resulted in an increased heart rate to 120 beats per minute. However, spontaneous breathing was inefficient and superficial, so the child was immediately intubated at the beginning of the second minute of life and was mechanically ventilated thereafter. 

Physical examination at birth found the infant to be eutrophic with a macrocephalic head. The sagittal suture was opened for 1.5 cm, and the large fontanel measured 5 × 6 cm while the small fontanel 1 × 2 cm. The abdomen was soft and the liver palpable at the right costal margin. Genitalia were extremely hypoplastic with barely indicated empty scrotum and a micropenis. Extremities appeared neat on the outside. Generalized hypotonia was observed with extremely rare spontaneous movements. 

**Figure 1:**
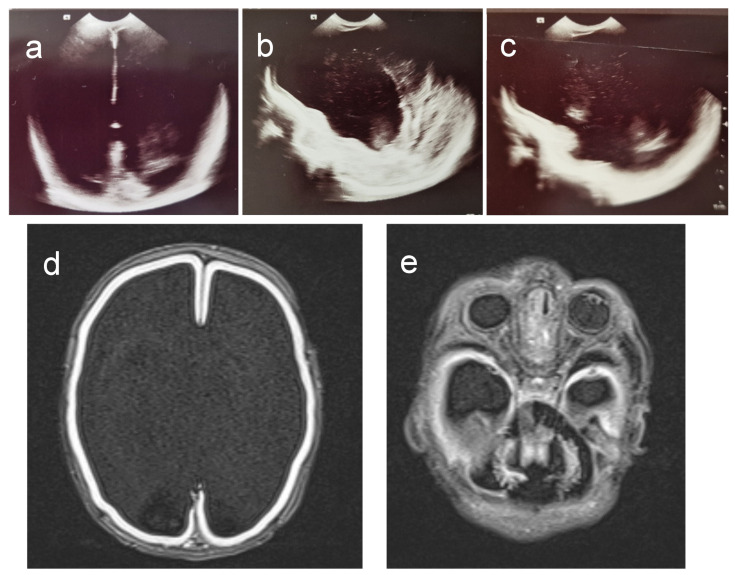
Brain ultrasound and MRI. (**a**–**c**). Brain ultrasound demonstrates dilated cerebral hemispheres, thin mantle of brain tissue on the convexity, absent basal ganglia and hypoplastic pons, and cerebellum. (**d**,**e**) Brain MRI showing complex anomalies consistent with WWS. (**a**) Coronary plane. (**b**) Medio-sagittal plane. (**c**) Parasagittal plane. (**d**) Supratentorial. (**e**) Infratentorial.

A brain ultrasound showed a medially positioned interhemispheric fissure. Cerebral hemispheres appeared dilated, filled with cerebrospinal fluid, and a thin mantle of the brain was visible on the brain convexity. The basal ganglia were not visible, nor were the choroid plexuses. The pons and cerebellum appeared hypoplastic (Figure 1a–c). Brain MRI showed an anomalous shape of all structures of the brainstem in the infratentorial area that morphologically shaped into the letter “Z”. The peduncles were extremely elongated and took on the shape of a “molar tooth sign”. The pons was flattened and folded ventrally. The vermis was not differentiated, and the cerebellar hemispheres were extremely hypoplastic. The fourth brain ventricle appeared voluminous and communicated with the mega cistern cerebellomedularis in terms of a Dandy Walker malformation/variant. Supratentorial, extremely hydrocephalically dilated lateral ventricles were visible, and the falx was differentiated in the ventral and dorsal parts. The cerebral hemispheres were reduced to a very narrow sheath of parenchyma, without the formation of sulci and gyri, suggesting a possible form of lissencephaly. Along the narrow parenchyma layer, marginally, in the presumed areas of the temporal horns, smaller, symmetrical crescent-shaped thickenings could be seen, which could correspond to lateralized and unconnected basal ganglia, i.e., rudimentary parts of the basal ganglia structures. In the sellar region, a very narrow Turkish saddle was observed. EEG activity showed pathological findings of diminished basic brain activity, suggestive of seizures. Echocardiography revealed patent atrial septal defect (ASD secundum) measuring 5 mm. The clinical presentation and complex congenital brain malformation (Figure 1d,e) indicated a Walker–Warburg Syndrome-like phenotype without eye anomalies. 

The patient was intubated at birth due to respiratory insufficiency and was mechanically ventilated during the entire stay. Empiric antibiotic therapy was prescribed. He developed generalized myoclonus, mainly during manipulation. Phenobarbitone and then midazolam was prescribed continuously until the twelfth day that led to diminishing of the motor manifestations. Meconium discharge was supported with glycerin suppository application, and oral tube feeding was practiced. Diuresis was established from the beginning; however, he became anuric on the seventh day despite optimal fluid intake. Diuresis was maintained with a dopamine infusion.

On day 15, pleuropneumonia due to *Staphylococcus warneri* was detected and treated with meropenem and vancomycin, as well as by transfusion of erythrocyte concentrates. On day 43, transfusion of erythrocytes was repeated. The infant died at 2 months of age from respiratory insufficiency and cardiopulmonary arrest. Genetic analysis was undertaken to look for a cause of the fatal infantile neurological disorder. 

### 3.2. Genetic Investigation

Chromosomal analysis of the infant showed a normal male karyotype of 46, XY. Whole-exome sequencing trio analysis of the patient and their biological parents was undertaken. We used slivar duo-del [13] to look for large deletions in the region of the exomes that arose de novo in the child. No large deletions were detected within the exome data. 

Small nucleotide variant (SNV) analysis was prioritized according to the likely inheritance models of de novo, X-linked, and recessive mutations (including compound heterozygotes). In our exome sequence analysis, a single nonsynonymous de novo heterozygous mutation was identified, p.His283Arg in *TUBA1A*: Chr12 (GRCh38): g.49185518T>C: NM_006009.4: c.848A>G, NP_006000.2:, as the most likely cause. We also manually examined the exome data for mutations in known WWS genes, OrphaNet: https://www.orpha.net/en/disease/gene/list/899 (accessed on 27 July 2024), and no causative variants were found.

**Figure 2:**
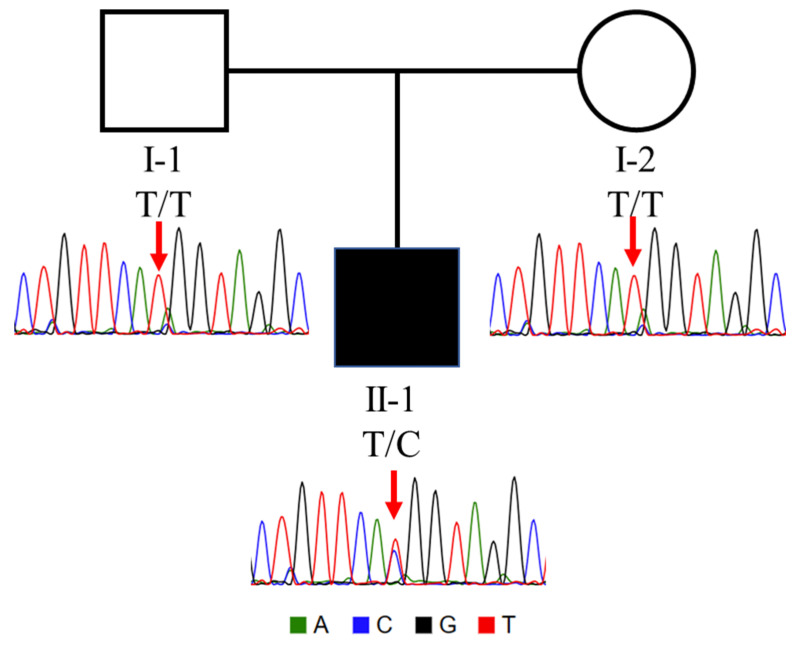
Trio pedigree with Sanger sequence chromatogram traces, showing the heterozygous *TUBA1A*, Chr12 (GRCh38): g.49185518T>C: NM_006009.4: c.848A>G mutation status in the patient (note that reverse sequencing trace is shown; thus, T>C).

**Figure 3:**
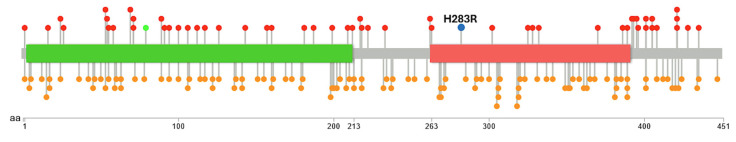
A lollipop diagram depicting the mutation landscape for TUBA1A variants reported in clinVar and the H283R mutation. TUBA1A (consists of two main functional domains represented by the green (Tubulin/FtsZ family, GTPase domain-amino acid (aa) 3-213) and red (Tubulin C-terminal domain-aa 263-392). Red upward lollipops represent pathogenic variants, orange downward lollipops represent likely pathogenic variants, a green upward lollipop represents a single likely benign variant, and the blue upward lollipop represents the H283R mutation identified in the patient.

The *TUBA1A* H283R mutation is extremely rare, appearing in only a single entry in clinvar (VCV001701083.1; rs2121243281), deposited by a clinical sequencing centre and listed as “likely pathogenic” with a single phenotypic descriptor of “seizure”. This entry of the mutation remains unreported in the literature and there are no assertation criteria provided (clinvar-0 stars). We performed direct Sanger sequencing of genomic DNA, which confirmed the presence of the mutation in the affected infant and the absence in the unaffected parents (Figure 2), confirming that the mutation was de novo. Initial InterVar [14] ACMG classification prediction for the mutation was adjusted from likely pathogenic (PM1 + PM2 + PP2 + PP3) to pathogenic (PS2 + PM1 + PM2 + PP2 + PP3); PS2 criteria were added based on Sanger validation confirmation (Figure 2). The PM1 criteria were supported by the absence of non-synonymous benign variants, and more than 10 pathogenic or likely pathogenic reported variants within the surrounding region (Figure 3) [20]. Multiple in silico predictions predicted that the mutation is damaging; SIFT (0.0), Polyphen2 (HVAR 0.929), MutationTaster (0.999), GERP++ (5.1), and REVEL (0.907). The CADD score of 25 strongly suggested pathogenicity. The c.848A>G mutation is in exon 4 of *TUBA1A* and is not present in the ALFA, ExAC, gnoMAD, and 1000 genomes genetic variant databases.

The mutation is located in the Tubulin C-terminal domain (Figure 3). We tabulate a summary of phenotypic information from the 46 pathogenic and likely pathogenic variants with phenotypic information in TUBA1A tubulin C-terminal domain, which are present in clinvar, and 24 of the same variants and their more detailed phenotypes as they were reported by Tantry et al. (2023) [16]) and our case (Table 2).

### 3.3. Analysis of Mutation Location in the 3D Structure of Tubulin α-1A Chain Protein in a Complex with β-Tubulin

**Figure 4:**
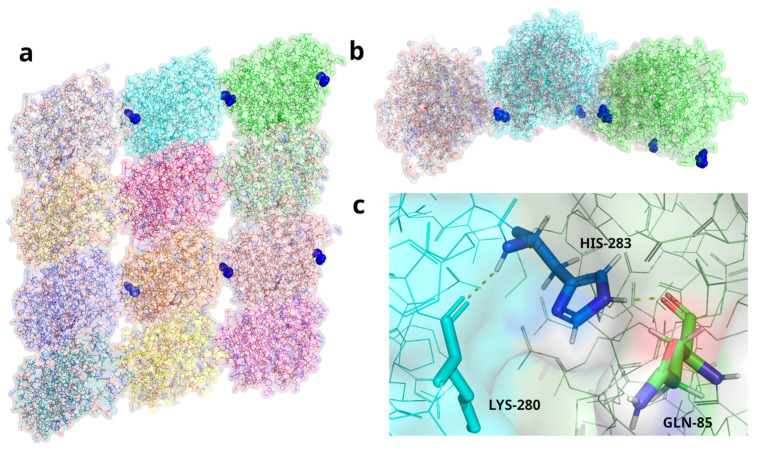
Three-dimensional modelling of TUBA1A Histidine (residue 283) in a neuronal microtubule filament structure. (**a**) Side view of neuronal human tubulin filament (modelled based on protein structure: 5JCO [13]). Alternating horizontal rows of α-tubulin 1A chain monomers (top row and third row) with dark blue spheres marking the H283 residue that facilitates interaction between adjacent α-tubulin 1A chain monomers. Each tubulin monomer is coloured differently to aid visualisation of interactions. Second and bottom rows are the tubulin-β-3 chain monomers of the microtubule filament. (**b**) Top view of microtubule filament shown in 4a, highlighting the location of His283 (dark blue spheres) lying deep in the groove between adjacent α-tubulin 1A chain monomers close to the internal surface of the microtubule filament. (**c**) Blow up of the structure of the His283 residue (dark blue) showing hydrogen bonding with Lys280 (turquoise) in the same α-tubulin 1A chain monomer and Gln85 (green) in the adjacent α-tubulin 1A chain monomer, which is predicted to be disrupted by the pHis283Arg mutation in the patient.

To explore our hypothesis of pathogenicity of the identified *TUBA1A* mutation, we sought to understand the possible effects of the His283Arg mutation on the structure of Tubulin α-1A Chain in a 3D structure of tubulin filaments. To do this, we performed protein visualization with PyMOL [17] on the published Cryo-EM structure of recombinant neuronal human tubulin 5JCO [18] to observe the Histidine 283 residue of interest and map its potential interactions. The location of the Histidine (residue 283) and the hydrogen bonds that it makes were marked on the α-Tubulin monomers in the 3D structure (Figure 4). Based on the published 3D Cryo-EM structure [18], the Histidine 283 residue is predicted to form two polar contacts: one as part of an innate backbone–backbone hydrogen bond with a neighbouring lysine at 280 (Lys280), and the second, an R-group, mediated polar contact through its imidazole ring with glutamine 85 (Gln85) of an adjacent α-tubulin subunit (Figure 4c). The second of these contacts would not be possible with α-tubulin that had an R-group substitution on residue 283 like the mutation of histidine to arginine found in our patient. This could cause a defect in lateral microtubule protofilament assembly, making it highly disruptive and, thus, pathogenic.

## 4. Discussion

*TUBA1A* encodes α-tubulin, a structural protein which plays a critical role in regulating neuronal migration in brain development [1]. Heterozygous de novo *TUBA1A* mutations cause an autosomal dominant “tubulinopathy”, with clinical features including brain malformations, microcephaly, neurodevelopmental delay, motor impairment, cognitive deficit, and epilepsy [1,21,22]. In this study, we identified a very rare *TUBA1A* mutation, His283Arg, located in the tubulin C-terminal domain (aa 263–392), in an infant that exhibited canonical tubulinopathy phenotypes with additional clinical features including some not previously reported with mutation of the gene.

*TUBA1A* is a highly constrained gene with very few missense mutations appearing in “healthy” population data. It has a Z-score of 8.75, indicating that it is very intolerant to amino acid changing variations without accompanying pathogenesis [23,24]. A total of 150 pathogenic and likely pathogenic variants with phenotypic information in *TUBA1A* are recorded in clinvar. Of these, almost a third (46) are in the tubulin C-terminal domain, between amino acids 263 and 392. In a recent review on the role of α tubulin isotypes in early brain development, Tantry et al. (2023) reported 77 published mutations, of which 24 were found in the same tubulin C-terminal domain [16]. The clinical features of mutations in this domain closely resemble those seen in our case. A comparison of phenotypic information for the tubulin C-terminal domain from our case, clinvar, and Tantry et al. (2023) is as shown in Table 2. 

In consistency with tubulinopathy cases in clinvar and Tantry et al. (2023), our case displayed features of lissencephaly, numerous brain malformations, and seizures/epilepsy. Unique to our case were the phenotypes of Dandy Walker malformation/ventricular dilation, macrocephaly, hypotonia, and hypoplastic genitalia with a micropenis. Hebebrand et al. (2019) observed that prenatally diagnosed fetal cases are more severe than those diagnosed after birth, which is consistent with our case, which was diagnosed in utero. 

As seen in Table 3, the majority of phenotypic features seen in our patient, except for macrocephaly, are also present in Walker–Walberg syndrome, which has the additional feature of retinal dysplasia. This overlap makes an accurate pre-term diagnosis very difficult without accompanying genetic analyses. 

Using the published 3D structure of neuronal tubulin [18], we visualized the location of residue 283, Histidine, in the repeating α-tubulin 1A chain monomers (Figure 4a,b). Based on the published 3D structure, the imidazole ring of Histidine 283 is predicted to interact with Glutamine (residue 85) on the adjacent α-tubulin monomer in the filament through hydrogen bonding (Figure 4c). This hydrogen bond is predicted to be disrupted when the Histidine is mutated to Arginine (p.H283R) in the patient. Disrupting this hydrogen bonding, we predict, will impair the lateral interaction and, thereby, binding between adjacent α-tubulin monomers. This 3D modelling and the extreme rarity of the mutations are highly suggestive of a high level of pathogenicity. 

Also, the novel clinical features seen in the infant are macrocephaly (rather than the more common microcephaly) and hypoplastic genitalia. The patient also exhibited a WWS-like generalized hypotonia, complex brain malformations, and seizures, which are also commonly seen in patients with *TUBA1A* mutations [2]. This infant was initially described as having clinical features resembling WWS, a severe form of autosomal recessive muscular dystrophy caused by mutations in glycosylation pathway genes. Clinical features of the syndrome include severe brain and eye abnormalities, as well as generalized severe hypotonia, muscle weakness, and seizures. Some of the clinical features overlap those associated with mutation of *TUBA1A*, highlighting the importance of determining a genetic diagnosis as early as possible (Table 3). 

## 5. Conclusions

The *TUBA1A* mutation identified in the patient, c.848A>G His283Arg, is de novo, being absent in both parents. The mutation is ultra rare in clinical databases and not present in control genetic databases, which indicate that it is most likely deleterious. Analyses using in silico prediction tools and ACMG guidelines classified the mutation as pathogenic. The mutation is in the tubulin C-terminal domain, which also contains 46 other pathogenic and likely pathogenic variants that are associated with similar patient phenotypes. Based on 3D modelling of the histidine 283 residue, in the α tubulin structure, H283R is predicted to form an R-group-mediated polar contact through its imidazole ring with glutamine 85 (Gln85) of an adjacent a-tubulin subunit. The mutation of the histidine residue to arginine, in our case, is postulated to disrupt this and, thereby, disrupt the intermolecular interactions between adjacent α-tubulin-1A chain monomers in the 3D structure of microtubule filaments. 

Together, the data support the *TUBA1A* mutation c.848A>G His283Arg as being causative of the neurological disorder and early death of the infant reported here. Genetic diagnosis of the patient allowed for genetic counselling to be offered to the family, including the risk of disease to other family members. Functional studies of the novel *TUBA1A* mutation, while challenging, may be warranted to determine the effects of the mutation on early brain development. Patients presenting with hypotonia, macrocephaly, and hypoplastic genitalia as well as brain malformations and seizures may not have WWS and should be considered for genetic testing for a possible tubulinopathy particularly involving the *TUBA1A* gene. 

## Figures and Tables

**Table 1:** Clinical features of affected patient. (N/A not applicable, + present).

**Patient Pedigree Number**	**II-1**
Sex	Male
Ethnicity	Croatian
Parental consanguinity	Non-consanguineous
**Clinical Features**	
Age at onset	Birth
Current age or age of death	Death at 2 months of age
Developmental delay or intellectual impairment	N/A
Gait abnormalities	N/A
Ataxia	N/A
Seizures	+
Neuropathy	N/A
Macrocephaly	+
Hypoplastic genitalia, micropenis	+
**cMRI Findings (affected regions)**	
Basal ganglia	+
Cortex	+
Corpus callosum	+
Cerebellum	+

**Table 2:** Count of variations/mutations reported in *TUBA1A* tubulin C-terminal domain (263–392) and associated phenotypical features. (+ clinical feature present).

Source/Database	ClinVar	Tantry et al. (2023) [16]	Case
Number of Mutations/Variations reported	46	24	1
**Phenotypes**			
Lissencephaly	+	+	+
Seizures/Epilepsy	+	+	+
Brain malformations (Cortex, Brain Stem)	+		
4th ventricle dilatation/Dandy Walker malformation/variant			+
Absence of language		+	
Anomalous structures of the brainstem			+
Autism		+	
Cerebellar hypoplasia		+	+
Congenital cataracts		+	
Corpus callosum agenesis			+
Hypotonia			+
Intellectual disability		+	
Macrocephaly			+
Microcephaly		+	
Microphthalmia		+	
Pachygyria		+	
Polymicrogyria		+	
Polymicrogyria-like		+	
Rudimentary and unconnected basal ganglia			+
Ventricular dilatation			+
Vermian aplasia (Cerebellar vermis aplasia)			+
Hypoplastic genitalia + micropenis			+

**Table 3:** Comparison of patient’s phenotypic characteristics with TUBA1A disorder and WWS syndrome.

TUBA1A Tubulinopathy	Patient	WWS
Microlysencephaly		
Lysencephaly	Lysencephaly	Lysencephaly
Polimicrogyria-like cortical dysplasia		
Corpus callosum agenesis	Corpus callosum agenesis	Corpus callosum hypoplasia/agenesis
Disorganized stiatum and thalami	Rudimentary and unconnected basal ganglia	
Cerebellar hypoplasia	Cerebellar hypoplasia	Cerebellar hypoplasia
Vermian hypoplasia	Vermian aplasia	Vermian hypoplasia
Brainstem hypoplasia	Anomalous structures of the brainstem	Flat brainstem
Ventricular dilatation	Ventricular dilatation	Ventricular dilatation
4th ventricle dilatation	4th ventricle dilatation/Dandy Walker malformation/variant	Dandy Walker malformation
Microcephaly	Macrocephaly	
Developmental delay	N/A	Developmental delay
Intellectual disability	N/A	Mental retardation
Seizures	Seizures	Seizures
Hypotonia	Hypotonia	Hypotonia
Eye malformations		Eye malformations
	Hypoplastic genitalia + micropenis	Undescended testes + micropenis
		Muscle weakness/congenital muscular dystrophy
		White matter hypomyelination
		Facial dysmorphic features

## Data Availability

The original contributions presented in this study are included in the article. All other data are available from the corresponding author on reasonable request.
